# Acquired von Willebrand syndrome is common in infants with systemic-to-pulmonary shunts: Retrospective case-series

**DOI:** 10.3389/fped.2022.1040128

**Published:** 2022-12-07

**Authors:** Vanya Icheva, Ulrich Budde, Harry Magunia, Karl Jaschonek, Clemens Hinterleitner, Felix Neunhoeffer, Christian Schlensak, Michael Hofbeck, Gesa Wiegand

**Affiliations:** ^1^ Department of Pediatric Cardiology and Intensive Care Medicine, University Children's Hospital Tübingen, Tübingen, Germany; ^2^ cMEDILYS Coagulation Lab mbH, Hamburg, Germany; ^3^ Department of Anesthesiology and Intensive Care Medicine, University Hospital Tübingen, Tübingen, Germany; ^4^ Department of Hematology, Oncology, Clinical Immunology and Rheumatology (Internal Medicine II), University Hospital Tübingen, Tübingen, Germany; ^5^ Department of Medical Oncology and Pneumology (Internal Medicine VIII), University Hospital Tübingen, Tübingen, Germany; ^6^ Department of Thoracic and Cardiovascular Surgery, University Hospital Tübingen, Tübingen, Germany

**Keywords:** von willebrand syndrome, infant, modified Blalock-Taussig-shunt, congenital heart defect, univentricular heart, systemic-to-pulmonary shunt

## Abstract

**Background:**

Although acquired von Willebrand syndrome (aVWS) has been described in congenital heart disease before, anatomical features leading to aVWS with characteristic reduction or loss of high molecular weight von Willebrand multimers (HMWM) are not well known. This study assesses the prevalence and effects of aVWS in infants with systemic-to-pulmonary shunts (SPS).

**Methods:**

This retrospective single-center study analyzes diagnostic data of infants with complex congenital heart defects requiring palliation with SPS. During the study period between 12/15–01/17 fifteen consecutive patients were eligible for analysis. Results of von Willebrand factor antigen (VWF:Ag), collagen binding activity (VWF:CB) and von Willebrand factor multimer analysis were included.

**Results:**

In all 15 patients with SPS an aVWS could be found. Blood samples were collected between 5 and 257 days after shunt implantation (median 64 days). None of the patients demonstrated increased bleeding in everyday life. However, 6 out of 15 patients (40%) showed postoperative bleeding complications after SPS implantation. Following shunt excision multimeric pattern normalized in 8 of 10 (80%) patients studied.

**Conclusions:**

This study shows that in patients undergoing SPS implantation aVWS might emerge. Pathogenesis can be explained by shear stress resulting from turbulent flow within the shunt. Knowledge of aVWS existence is important for the consideration of replacement therapy with von Willebrand factor containing products and antifibrinolytic treatment in bleeding situations. Implementation of methods for rapid aVWS detection is required to achieve differentiated hemostatic therapy and reduce the risk of complications caused by empiric replacement therapy.

## Introduction

Von Willebrand factor (VWF) is an important glycoprotein in primary hemostasis. It is produced by endothelial cells and megakaryocytes and released as high molecular weight von Willebrand multimers (HMWM) (1). It circulates in the blood plasma in complex with factor VIII, which is thereby protected from proteolysis. It can bind to proteins of the subendothelial matrix as well as to the von Willebrand receptor (glycoprotein Ib/IX) on the surface of platelets. Thus, as a so-called adhesive protein, it creates a connection between the platelets and the injured vessel wall and activates the platelets (2).

Acquired von Willebrand syndrome (aVWS) is a bleeding diathesis, which has been described occasionally in children with congenital heart defects (CHD), such as aortic and pulmonary stenosis (3–5), persisting arterial duct (PDA) (6) and ventricular septum defect (VSD) (7). These specific hemodynamic profiles are associated with increased shear stress, which can induce aVWS (8). Shear stress is responsible for increased unfolding of the HMWM, which subsequently undergo ADAMTS 13 mediated proteolysis within their mechanosensitive A2 domain (9). This results in a significant decrease or loss of HMWM when the shear stress source is persisting.

The VWF multimer analysis is the gold standard test for diagnosing aVWS. However this test is only available in specialized laboratories (10). In clinical practice additional parameters testing VWF function, such as the ratio of ristocetin cofactor activity VWF:RCo/VWF:Ag and collagen binding VWF:CB/VWF:Ag can be used as an indicator of aVWS (11). Despite the availability of these tests, they have not been proved as reliable diagnostic tools (10, 12). This makes the diagnosis challenging and can lead to underestimation of aVWS incidence in pediatric patients with CHD (13).

In a previous study we found that aVWS can be present in the perioperative course of up to 80% of neonates, undergoing heart surgery for complex CHD (14).

AVWS often remains unrecognized in everyday life, since patients usually don't have any spontaneous bleeding episodes, but they can encounter increased bleeding during surgery or trauma (15). AVWS has also been shown to be a relevant cause for major bleeding in children treated on pediatric cardiac intensive care units (16).

Surgical palliation in neonates with functionally univentricular hearts is complex and frequently includes creation of systemic-to-pulmonary artery shunts (SPS) followed by cardiac catheterizations and further surgical procedures. A variety of SPS have been introduced, including modified Blalock-Taussig-Shunts (BTS), the Sano-Shunt and central aortopulmonary shunts. Their common feature is an accelerated and turbulent blood flow across the prosthetic shunt due to the marked difference between systemic and pulmonary vascular pressure, thus theoretically generating conditions for HMWM consumption under high shear stress.

Several studies have confirmed the significantly increased incidence of bleeding and thrombotic complications in this group of patients (17, 18). To the best of our knowledge, there are no reports describing the von Willebrand status in infants with SPS so far. The objective of this study was to describe the prevalence and time course of aVWS in infants requiring SPS as part of the palliation of complex CHD.

## Materials and methods

### Study design and setting

**Figure 1:**
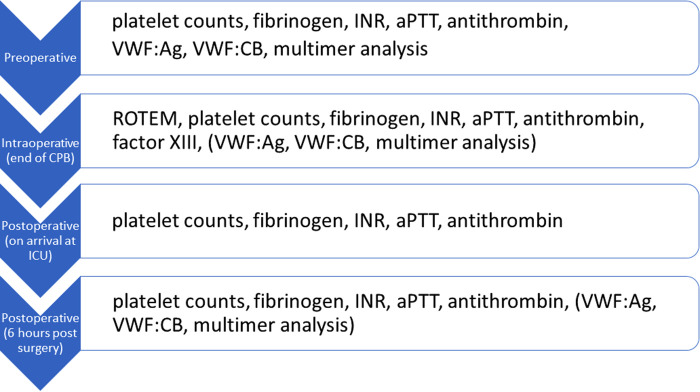
Workflow of departmental coagulation monitoring.

This single-center, retrospective, observational study assesses the clinical and laboratory manifestation of aVWS in infants with functionally univentricular hearts following creation of a SPS. All patients were treated at our tertiary referral center from December 2015 to January 2017. Laboratory tests, including perioperative and periinterventional screening for aVWS, routinely performed as part of the coagulation monitoring after SPS establishment, were included in this analysis. Figure 1 shows the workflow of our coagulation monitoring. VWF diagnostics were routinely performed before cardiac catheterization or preoperatively. Postoperative analysis of VWF values was possible at the discretion of the treating physicians.

### Patient selection and data collection

Data collection was performed retrospectively based on the medical charts of all children with already created SPS undergoing cardiac catheterization or surgery in the period mentioned above. Inclusion criteria were the diagnosis of complex CHD and the presence of SPS. Results of coagulation tests and von Willebrand multimer analyses were extracted from the medical charts. Patients with missing laboratory data on VWF were excluded. The collected data included echocardiography findings, laboratory results, data on blood component therapy as well as bleeding and thromboembolic events in everyday life and during heart catheterizations or operations.

The patients' characteristics, including the diagnoses, the shunt type and the aVWS-status at different stages (follow up) are summarized in Table 1. All pressure gradients were determined by Doppler echocardiography and represent the highest systolic peak instantaneous gradient. Coagulation therapy during surgeries was guided by a ROTEM-based algorithm. Postoperative anticoagulation after shunt implantation was performed with unfractionated heparin in therapeutic doses. After removal of the central line, heparin was switched to aspirin and clopidogrel in all patients with SPS. For planned cardiac catheterization or surgery, aspirin and clopidogrel were paused and bridging with unfractionated heparin was performed.

**Table 1:** Patients’ characteristics and aVWS status.

Patient Number	Diagnosis	Shunt-Type	aVWS-Status	Follow-up (I): Surgery with Shunt Excision	Intraoperative aVWS-Monitoring	aVWS-Status (I)	Follow-up (II): Time to Blood Sampling after Surgery	aVWS-Status (II)
1.	HLHS	BTS	++		Lost to follow up			
2.	PAtr with IVS	CAPS	++		Lost to follow up			
3.	TA IIc	BTS	+	Glenn	After CPB discontinuation	-	6h post CPB	-
4.	HLHS	BTS	++	Glenn	No intraoperative monitoring		70 days	-
5.	Critical PSt, hypoplastic RV	BTS	++	Died before Glenn				
6.	HLHS	BTS	++	Died before Glenn				
7.	TA Ic	BTS	++	Glenn	After CPB discontinuation	++	4 months	-
8.	HLHS	BTS	+	Glenn	After CPB discontinuation	+	6h post CPB	-
9.	HLHS	Sano Shunt	++	Glenn	After CPB discontinuation	+	6h post CPB	-
10.	HLHS	BTS	+		Lost to follow up			
11.	TA IIb	BTS (2x)	++	Glenn	After CPB discontinuation	-	20h post CPB	-
12.	UH, Right isomerism, PSt	BTS	++	Glenn	After CPB discontinuation	++	6h post CPB	-
13.	HLHS, Bipulmonary banding	BTS	++	Glenn	After CPB discontinuation	+	6 h post CPB	++
14.	PAtr with VSD (biventricular)	BTS	++	VSD-closure and RVOT-PA-Patch	No intraoperative monitoring		13 days	+
15.	PAtr with VSD	BTS	++	RV-PA-Conduit, VSD- Patch-Fenestration	6 days after BTS excision and establishment of RV-PA-Conduit	++	6 months after RV-PA-Conduit	-

aVWS, Acquired von Willebrand Syndrome, BTS, modified Blalock-Taussig-Shunt; CAPS, Central Aortopulmonary Shunt, CPB, Cardiopulmonary Bypass, DILV, Double Inlet Left Ventricle, HLHS, Hypoplastic Left Heart Syndrome, IVS, Intact Ventricular Septum, PA, Pulmonary Artery, PAtr, Pulmonary Atresia; PDA, Patent Ductus Arteriosus; PSt, Pulmonary Valve Stenosis; RV, Right Ventricle; RVOT, Right Ventricular Outflow Tract; TA, Tricuspid Atresia; UH, Univentricular Heart; VSD, Ventricular Septal Defect; - (negative or eliminated), + (moderate aVWS), ++ (severe aVWS).

### Laboratory analysis

Von Willebrand antigen (VWF:Ag), collagen binding (VWF:CB) and Willebrand multimers were analyzed in an external reference lab (cMEDILYS Coagulation Lab mbH, Hamburg, Germany) as described previously (19, 20). The aVWS was graded as severe (++) if either a complete loss of the largest multimers was present or if the high molecular multimers were less than 20% of all multimers. Moderate aVWS (+) was defined as a relative loss of the high molecular multimers resulting in a disproportion between the high and low molecular multimers.

### Statistical analysis

All statistical analyses as well as figure design were performed using GraphPad Prism 8 (Version 8.4.0 for Windows, GraphPad Software, Inc.). Continuous data are expressed as median and interquartile range. HMWM values before and after shunt excision were analyzed with regard to significant differences by the Wilcoxon matched-pairs signed rank test. Correlations between HMWM values and echocardiographic gradients as well as sampling time were tested by means of linear regression analysis. A probability of *p* < 0.05 was defined as significant for all statistical tests.

## Results

### Patient characteristics

During the study period 17 children with complex CHD and SPS were screened for possible inclusion in our analysis. Two children were excluded because no VWF testing after SPS implantation had been performed. Fifteen patients aged 3–14 months (median 4 months) with complex congenital heart disease following SPS surgery could be included in the analysis. The median patient weight was 5.6 (3.5–8.5) kg at the sampling.

The blood tests for aVWS were performed between day 5 to 257 after shunt implantation surgery (median 64 days), usually before elective cardiac catheterisation procedures or SPS excision surgery. Shunt types included 13 modified Blalock-Taussig-Shunts (BTS), 1 central aortopulmonary shunt, and 1 Sano-shunt. All patients were in a stable clinical condition at the time of blood sampling.

In 10 of the 15 patients, VWF parameters were also measured after SPS excision on the discretion of the attending physicians. The SPS excisions were performed either as part of the Glenn surgery (8 patients) or during biventricular repair (2 patients). Timing of follow-up blood sampling varied from intraoperatively to 6 months postoperatively. The intraoperative tests for aVWS were performed in 7 patients immediately after discontinuation of cardiopulmonary bypass (CPB) and before treatment with blood components. Details on timing of blood sampling are listed in Table 1.

Of the patients who were not followed up, one moved and two deceased (one suspected acute shunt thrombosis and one acute onset 3rd grade atrioventricular block). In two cases, no follow-up sampling after SPS excision was performed.

### Prevalence of aVWS at stage systemic-to-pulmonary artery shunt

In all 15 shunt-patients a reduction or loss of HMWM could be detected after creation of SPS. Thirteen patients had a severe form of aVWS and 2 patients had moderate aVWS. Only one patient (No. 14) was initially tested negative for aVWS on day 5 after the shunt implantation. At this time an echocardiographic gradient of 72 mmHg across the BTS was detectable. The same patient tested positive for severe aVWS on day 34 after shunt creation. The other patients were tested on the 17th postoperative day or later and all of them had aVWS on multimer analysis.

**Figure 2:**
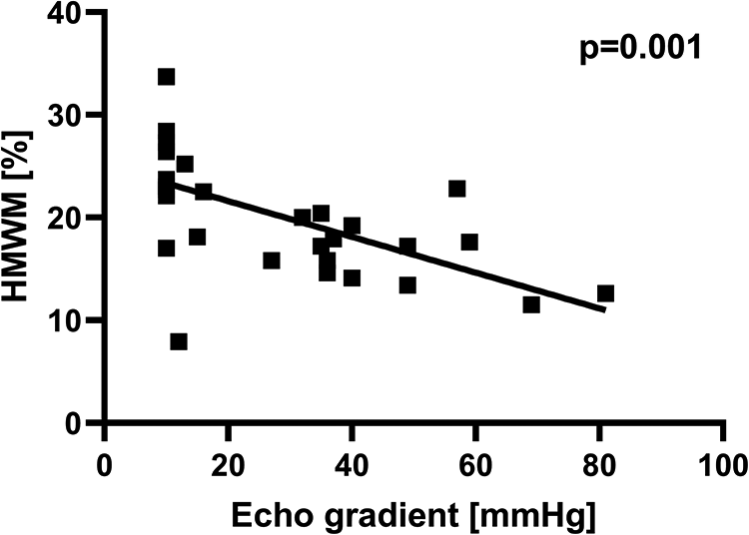
HMWM decrease was related significantly to peak systolic echocardiographic gradients.

The peak echocardiographic pressure gradients across the shunt ranged between 27 to 81 mmHg at the time of multimer testing. The HMWM level was inversely related to the estimated peak systolic gradient (Figure 2).

The severity of HMWM reduction did not correlate significantly with the time interval after shunt implantation.

### Prevalence of aVWS following excision of the systemic-to-pulmonary artery shunt

In the available follow up samples of 10 patients the VWF:CB/VWF:Ag ratio increased in all of them compared with the preoperative values.

**Figure 3:**
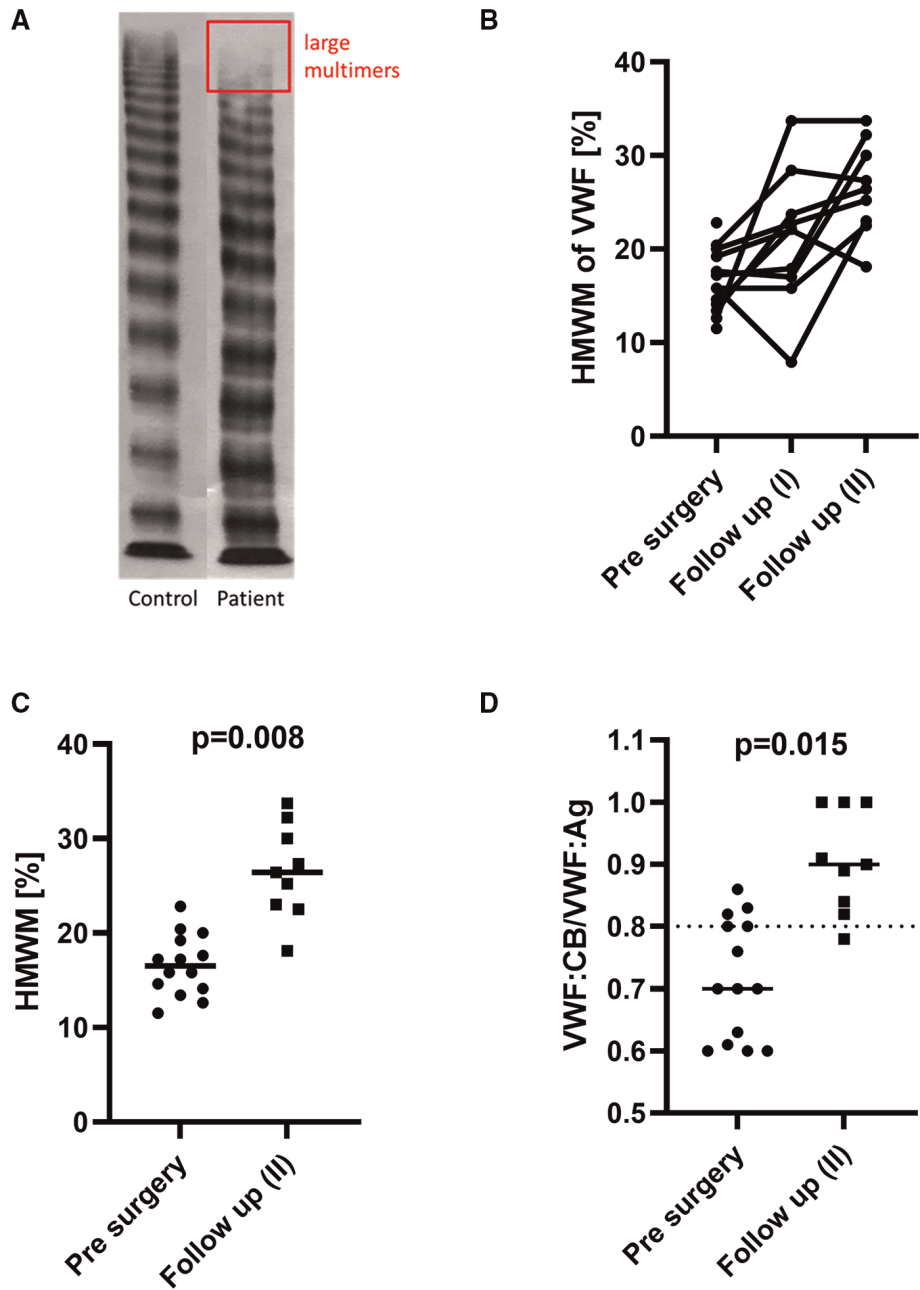
An exemplary multimer analysis (**A**) of the plasma of one shunt patient (No. 11) with a complete loss of the largest multimers (the rectangle corresponds to the typical area of distribution of HMWM on gel electrophoresis; the full gel was cropped for the sake of clarity). The individual course of every patient is presented in (**B**) and shows gradual HMWM recovery following shunt excision surgery for most patients. The HMWM values (**C**) and also the ratio VWF:CB/VWF:Ag (**D**) increased significantly after shunt excision.

The multimeric pattern normalized in 8 of the patients. No relevant residual stenosis was found in any of them on echocardiography. In the remaining 2 patients, the percentage of HMWM increased, but moderate aVWS persisted throughout the follow-up period after shunt excision surgery (Figure 3). One of them (No. 13) received Glenn surgery and the other one (No. 14) received biventricular correction of pulmonary atresia and VSD. Patient No. 13 had multiple aortopulmonary collaterals as possible source of shear stress. Patient No. 14 had only a mild residual stenosis of the left pulmonary artery after surgery.

In addition, one patient (No. 15) was found to have severe aVWS and significant residual pulmonary bifurcation obstruction after corrective surgery. AVWS resolved on repeat testing after treatment of the obstruction by interventional stent placement.

Specifically, intraoperative screening for aVWS was performed in 7 patients after discontinuation of CPB. At this time, the multimeric pattern had improved in 2 of them and completely normalized in another 2 patients.

### Clinical course

Bleeding complications were observed mainly in the early postoperative course after shunt implantation (up to day 7). Six of 15 patients experienced thoracic hemorrhages, requiring surgical revision. However, four of the six bleeding episodes occurred during ECMO therapy. The other two cases were non-surgical diffuse thoracic hemorrhages requiring revision. AVWS tests from the time of the acute hemorrhage were not available. None of the shunt patients had spontaneous bleeding in everyday life. No clinically observed increased bleeding occurred during cardiac catheterizations. The majority of patients (12 of 15) yet received elective red blood cell (RBC) transfusions after their cardiac catheterization procedures because of post interventional drop of hemoglobin values. Concerning the operative shunt excision, usually as a part of the Glenn operation, no severe perioperative bleeding events were observed.

Two of the patients also experienced major thromboembolic complications during the observation period. Patient No. 4 experienced acute shunt thrombosis on the day of shunt placement. Patient No. 6 developed a thrombosis of the superior vena cava on the 10th postoperative day and died of acute shunt thrombosis in the further course.

## Discussion

To the best of our knowledge this is the first report describing the prevalence of aVWS among patients following creation of SPS. AVWS was detected in all 15 analyzed infants. Despite the relatively small number of our cohort, we can presume that SPS are associated with aVWS in the majority of cases. This finding is remarkable but also to some extent expected, since establishment of a palliative SPS results in high-flow, high-velocity artificial vessels that are likely to generate enough shear stress to cause damage to the large von Willebrand multimers.

This is also supported by the fact that aVWS recovered completely in 8 of 10 patients tested after shunt excision. The HMWM pattern improved in the remaining 2 patients.

Shunt palliation procedures (e.g., Norwood procedure) in neonates with complex CHD are associated with a high rate of perioperative bleeding, as our data show. The causes are multifactorial and include surgical problems, various coagulation disorders, need for anticoagulation and possible extracorporeal membrane oxygenation (ECMO) therapy (21). Whether and to what extent aVWS contributes to intraoperative and early postoperative bleeding complications cannot be determined from our data, because only one patient in our cohort was tested in this period and did not have aVWS, which, however, developed during the course. This indicates that aVWS may evolve with some delay after surgery. A possible explanation could be increasing shear stress and pressure gradient across the shunt due to increasing relative shunt stenosis in the growing patient, which was already hypothesized by Rannuci et al. (22).

AVWS has previously been shown to be associated with higher peak velocity gradients and is a potential cause of increased bleeding during invasive procedures in infants with complex CHD requiring palliative surgery (23). Recent studies show that treatment algorithms including supplementation of VWF concentrate may reduce perioperative blood loss in children with congenital heart disease (24). Other treatment options such as desmopressin (DDAVP) administration are not very effective in cardiovascular aVWS because patients have high levels of circulating VWF with impaired function (10), and they do not reduce bleeding or transfusion requirements in congenital heart surgery (25). However, neonates and infants with prosthetic shunts are also at increased risk of life-threatening thromboembolic complications due to a CHD-associated imbalance between pro- and antithrombotic activity, abnormally low blood flow velocities and prothrombotic foreign materials (26–32). This was also evident in our case series, as 2 patients died from shunt thrombosis. Hunt et al. described lower postoperative levels of ADAMTS13 in the perioperative period of neonates and infants with CHD (33), which should be taken into account when considering VWF concentrate supplementation. In this context, the introduction of additional routine laboratory parameters such as the VWF:GPIbM/VWF:Ag ratio is a promising possibility for a timely aVWS diagnosis in the perioperative course of paediatric CHD patients (34). In our opinion, implementation of methods for rapid aVWS detection is necessary and should be available in centers performing neonatal cardiac surgery and providing extracorporeal support. The introduction of this new component into existing diagnostic and bleeding treatment algorithms has the potential to further reduce the risk of thromboembolic complications caused by possible empirical replacement therapy with VWF concentrates or other proven thrombogenic substances such as rFVIIa (35).

So far none of our patients demonstrated an increased bleeding tendency in everyday life, as spontaneous bleeding events are not typical for this condition (13). The effects on bleeding symptoms during minor surgery or cardiac catheterisation also appear to be limited and well manageable without specific aVWS treatment.

The patients in our cohort had no clinically observed increased bleeding during cardiac catheterisation procedures or shunt excision surgery (usually the Glenn procedure). It is unclear if aVWS contributed to blood loss during catheterisation procedures since also other factors, such as challenging vessel puncture, anticoagulation and repeated blood sampling can cause drop of hemoglobin. However, if significant bleeding episodes cannot be explained otherwise, aVWS should be considered as a possible cause in shunt patients. To exclude typical reasons for a drop in hemoglobin, a prospective comparison of patients with and without aVWS after cardiac catheterization might be useful in future studies.

After shunt excision the HMWM recovered quite rapidly and this might be one of the reasons why postoperative bleeding complications were rare. However, other causes, such as the shorter CPB time, the smaller wound area, and the older age of the patients compared to the SPS implantation surgery, may have also contributed to the lower bleeding rate.

Our study has several limitations. Since our shunt patients were aVWS positive, it was not possible to determine if the aVWS-positive patients have a higher bleeding tendency as compared to aVWS-negative patients during invasive procedures. Data on aVWS status during acute hemorrhage in the early postoperative period after SPS establishment were not available. AVWS tests prior to surgical or catheter interventions were dependent on the timing of these procedures. Thus, the time point of blood sampling varied considerably between patients. In addition, there were large differences in the underlying pathology of the patients, which cannot be compensated for due to the lack of a non-shunt control group. Follow-up tests were only performed according to the decision of the attending physicians. Confounders like treatment with fresh frozen plasma or influence of CPB during surgery cannot clearly be excluded for the results of early postoperative sampling after SPS excision. The study population did not include all consecutive patients who underwent shunt operations during the study period (two patients were excluded due to incomplete laboratory data). Finally, the potential risks of thromboembolic complications from treatment with VWF concentrates in this patient population have not yet been investigated and should be addressed in future studies.

## Conclusions

In conclusion, aVWS is a phenomenon occurring in the majority of infants with SPS. Its contribution to increased bleeding during invasive procedures has not yet been sufficiently elucidated and needs to be further investigated in larger controlled prospective trials. The intention of this report is to raise the awareness of treating physicians for aVWS as a possible cause of hemorrhage during or after invasive procedures in this subgroup of patients. In addition, attention should be drawn to existing rapid diagnostic tests as well as therapy with von Willebrand factor-containing products and antifibrinolytic treatment. However, data on safety and efficacy are still limited and need to be a subject of further controlled trials.

## Data Availability

The raw data supporting the conclusions of this article will be made available by the authors, without undue reservation.

## References

[1] Federici AB Budde U Castaman G Rand JH Tiede A. Current diagnostic and therapeutic approaches to patients with acquired von Willebrand syndrome: a 2013 update. Semin Thromb Hemost. (2013) 39(2):191–201. 10.1055/s-0033-1334867 23397553

[2] Bryckaert M Rosa JP Denis CV Lenting PJ. Of von Willebrand factor and platelets. Cell Mol Life Sci. (2015) 72(2):307–26. 10.1007/s00018-014-1743-8 25297919 PMC4284388

[3] Wiegand G Hofbeck M Zenker M Budde U Rauch R. Bleeding diathesis in Noonan syndrome: is acquired von Willebrand syndrome the clue? Thromb Res. (2012) 130(5):e251–4. 10.1016/j.thromres.2012.08.314 22985731

[4] Vincentelli A Susen S Le Tourneau T Six I Fabre O Juthier F Acquired von Willebrand syndrome in aortic stenosis. N Engl J Med. (2003) 349(4):343–9. 10.1056/NEJMoa022831 12878741

[5] Sadler JE. Aortic stenosis, von Willebrand factor, and bleeding. N Engl J Med. (2003) 349(4):323–5. 10.1056/NEJMp030055 12878737

[6] Rauch R Budde U Koch A Girisch M Hofbeck M. Acquired von Willebrand syndrome in children with patent ductus arteriosus. Heart. (2002) 88(1):87–8. 10.1136/heart.88.1.87 12067958 PMC1767161

[7] Gill JC Wilson AD Endres-Brooks J Montgomery RR. Loss of the largest von Willebrand factor multimers from the plasma of patients with congenital cardiac defects. Blood. (1986) 67(3):758–61. 10.1182/blood.V67.3.758.758 3484979

[8] Heilmann C Geisen U Beyersdorf F Nakamura L Benk C Trummer G Acquired von Willebrand syndrome in patients with extracorporeal life support (ECLS). Intensive Care Med. (2012) 38(1):62–8. 10.1007/s00134-011-2370-6 21965100

[9] Lippok S Radtke M Obser T Kleemeier L Schneppenheim R Budde U Shear-Induced unfolding and enzymatic cleavage of full-length VWF multimers. Biophys J. (2016) 110(3):545–54. 10.1016/j.bpj.2015.12.023 26840720 PMC4744175

[10] Budde U Scheppenheim S Dittmer R. Treatment of the acquired von Willebrand syndrome. Expert Rev Hematol. (2015) 8(6):799–818. 10.1586/17474086.2015.1060854 26577336

[11] Tiede A Priesack J Werwitzke S Bohlmann K Oortwijn B Lenting P Diagnostic workup of patients with acquired von Willebrand syndrome: a retrospective single-centre cohort study. J Thromb Haemost. (2008) 6(4):569–76. 10.1111/j.1538-7836.2008.02909.x 18208537

[12] Budde U Pieconka A Will K Schneppenheim R. Laboratory testing for von Willebrand disease: contribution of multimer analysis to diagnosis and classification. Semin Thromb Hemost. (2006) 32(5):514–21. 10.1055/s-2006-947866 16862525

[13] Avila ML Lee KJ Bouskill V Rand ML James P Carcao M. Acquired von Willebrand syndrome in paediatric patients with congenital heart disease: challenges in the diagnosis and management of this rare condition. Haemophilia. (2015) 21(1):e89–92. 10.1111/hae.12567 25495773 PMC4675139

[14] Icheva V Nowak-Machen M Budde U Jaschonek K Neunhoeffer F Kumpf M Acquired von Willebrand syndrome in congenital heart disease surgery: results from an observational case-series. J Thromb Haemost. (2018) 16(11):2150–8. 10.1111/jth.14208 29908036

[15] Stockschlaeder M Schneppenheim R Budde U. Update on von Willebrand factor multimers: focus on high-molecular-weight multimers and their role in hemostasis. Blood Coagul Fibrinolysis. (2014) 25(3):206–16. 10.1097/MBC.0000000000000065 24448155 PMC3969155

[16] Jones MB Ramakrishnan K Alfares FA Endicott KM Oldenburg G Berger JT Acquired von Willebrand syndrome: an under-recognized cause of Major bleeding in the cardiac intensive care unit. World J Pediatr Congenit Heart Surg. (2016) 7(6):711–6. 10.1177/2150135116658011 27834763

[17] Agarwal A Firdouse M Brar N Yang A Lambiris P Chan AK Incidence and management of thrombotic and thromboembolic complications following the superior cavopulmonary anastomosis procedure: a literature review. Clin Appl Thromb Hemost. (2018) 24(3):405–15. 10.1177/1076029617739702 29277101 PMC6714653

[18] Shinkawa T Holloway J Tang X Gossett JM Imamura M. Experience using kaolin-impregnated sponge to minimize perioperative bleeding in Norwood operation. World J Pediatr Congenit Heart Surg. (2017) 8(4):475–9. 10.1177/2150135117713698 28696876

[19] Budde U Schneppenheim R Eikenboom J Goodeve A Will K Drewke E Detailed von willebrand factor multimer analysis in patients with von willebrand disease in the European study, molecular and clinical markers for the diagnosis and management of type 1 von willebrand disease (MCMDM-1VWD). J Thromb Haemost. (2008) 6(5):762–71. 10.1111/j.1538-7836.2008.02945.x 18315556

[20] Hinterleitner C Kreisselmeier KP Pecher AC Mauz PS Kanz L Kopp HG Low plasma protein Z levels are associated with an increased risk for perioperative bleedings. Eur J Haematol. (2018) 100(5):403–11. 10.1111/ejh.13031 29360177

[21] Wang SG Griffith BP Wu ZJJ. Device-Induced hemostatic disorders in mechanically assisted circulation. Clin Appl Thromb-Hem. (2021) 27:1–14. 10.1177/1076029620982374 PMC7883139 33571008

[22] Ranucci M Giamberti A Baryshnikova E. Is there a role for von Willebrand factor/factor VIII concentrate supplementation in complex congenital heart surgery? J Thromb Haemost. (2018) 16(11):2147–9. 10.1111/jth.14268 30112830

[23] Loeffelbein F Funk D Nakamura L Zieger B Grohmann J Siepe M Shear-stress induced acquired von Willebrand syndrome in children with congenital heart disease. Interact Cardiovasc Thorac Surg. (2014) 19(6):926–32. 10.1093/icvts/ivu305 25228244

[24] Wolf J Brandenburger C Dittrich M Flieder T Koster A Budde U Treatment algorithm for patients with von willebrand syndrome type 2A and congenital heart disease-A treatment algorithm may reduce perioperative blood loss in children with congenital heart disease. Pediatr Crit Care Med. (2022) 23(10):812–21. 10.1097/PCC.0000000000003026 35834676

[25] Oliver WC Jr. Santrach PJ Danielson GK Nuttall GA Schroeder DR Ereth MH. Desmopressin does not reduce bleeding and transfusion requirements in congenital heart operations. Ann Thorac Surg. (2000) 70(6):1923–30. 10.1016/S0003-4975(00)02176-7 11156096

[26] Todd Tzanetos DR Yu C Hernanz-Schulman M Barr FE Brown NJ. Prospective study of the incidence and predictors of thrombus in children undergoing palliative surgery for single ventricle physiology. Intensive Care Med. (2012) 38(1):105–12. 10.1007/s00134-011-2378-y 21979273 PMC4747610

[27] Heying R van Oeveren W Wilhelm S Schumacher K Grabitz RG Messmer BJ Children undergoing cardiac surgery for complex cardiac defects show imbalance between pro- and anti-thrombotic activity. Crit Care. (2006) 10(6):R165. 10.1186/cc5108 17125503 PMC1794476

[28] Eaton MP Iannoli EM. Coagulation considerations for infants and children undergoing cardiopulmonary bypass. Paediatr Anaesth. (2011) 21(1):31–42. 10.1111/j.1460-9592.2010.03467.x 21155925

[29] Cholette JM Rubenstein JS Alfieris GM McDermott MP Harmon WG Vermilion R Elevated risk of thrombosis in neonates undergoing initial palliative cardiac surgery. Ann Thorac Surg. (2007) 84(4):1320–5. 10.1016/j.athoracsur.2007.05.026 17888990

[30] Emani S Zurakowski D Baird CW Pigula FA Trenor C 3rd Emani SM. Hypercoagulability panel testing predicts thrombosis in neonates undergoing cardiac surgery. Am J Hematol. (2014) 89(2):151–5. 10.1002/ajh.23607 24123221

[31] Bockeria LA Samsonova NN Yurlov IA Klimovich LG Kozar EF Olsen EH Dynamics of factor XIII levels after open heart surgery for congenital heart defects: do cyanotic and acyanotic patients differ? Pediatr Cardiol. (2014) 35(7):1108–15. 10.1007/s00246-014-0903-9 24714980

[32] Mascio CE Irons ML Ittenbach RF Gaynor JW Fuller SM Kaplinski M Thirty years and 1663 consecutive Norwood procedures: has survival plateaued? J Thorac Cardiovasc Surg. (2019) 158(1):220–9. 10.1016/j.jtcvs.2018.12.117 31248509

[33] Hunt R Hoffman CM Emani S Trenor CC 3rd Emani SM Faraoni D Elevated preoperative von Willebrand factor is associated with perioperative thrombosis in infants and neonates with congenital heart disease. J Thromb Haemost. (2017) 15(12):2306–16. 10.1111/jth.13860 28981194

[34] Icheva V Ebert J Budde U Wiegand G Schober S Engel J Perioperative diagnosis and impact of acquired von Willebrand syndrome in infants with congenital heart disease. Blood. (2022) 10.1182/blood.2022015699. [Epub ahead of print] 36054926

[35] Gill R Herbertson M Vuylsteke A Olsen PS von Heymann C Mythen M Safety and efficacy of recombinant activated factor VII: a randomized placebo-controlled trial in the setting of bleeding after cardiac surgery. Circulation. (2009) 120(1):21–7. 10.1161/CIRCULATIONAHA.108.834275 19546387

